# Predictors of Infrazygomatic Crest Implant Failure: A Prospective Study on Risk Factors, Stability, and Clinical Outcomes in Orthodontic Anchorage

**DOI:** 10.7759/cureus.81862

**Published:** 2025-04-08

**Authors:** Prachi Pragya, Khadeer Riyaz, Vikram Karande, Vishwapratapsingh Chavan, Rohit K Shinde, Priyanka Medhi, Seema Gupta

**Affiliations:** 1 Department of Orthodontics and Dentofacial Orthopedics, Indira Gandhi Institute of Medical Sciences, Patna, IND; 2 Department of Orthodontics and Dentofacial Orthopedics, The Oxford Dental College and Research Centre, Bangalore, IND; 3 Department of Oral and Maxillofacial Surgery, D Y Patil Dental School, Pune, IND; 4 Department of Orthodontics and Dentofacial Orthopedics, Kothiwal Dental College and Research Centre, Moradabad, IND

**Keywords:** factors, failure, implants, orthodontic, risk

## Abstract

Introduction: Infrazygomatic crest (IZC) implants are widely used for orthodontic anchorage. However, their success is influenced by multiple factors. This study aimed to evaluate the failure rates of IZCs and identify key predictors, including age, sex, implant placement characteristics, loading protocols, inflammation, mobility, bone density, and oral hygiene. Understanding these factors can help optimize treatment strategies and improve implant survival rates.

Materials and methods: This prospective, cross-sectional observational study was conducted at the Department of Orthodontics from August 2022 to December 2024. Sixty patients requiring IZC implants were enrolled, with the systematic exclusion of those with systemic conditions affecting bone metabolism. The implants (Dentos Absoanchor, 12/14 mm length, 2 mm diameter) were placed at an insertion angle of 0-90^0^ under standardized surgical protocols. Bone density was assessed using cone-beam computed tomography (CBCT), and the patients were divided into immediate and delayed loading groups. Implant failure was defined as loss or removal within eight months of placement. Statistical analyses included univariate regression, receiver operating characteristic (ROC) curve analysis, and odds ratio (OR) calculations.

Results: Of 60 implants, 18 (30%) failed. Patients aged > 18 years had a higher failure rate (~35%) than those aged < 18 years (~25%), while females exhibited a greater failure rate than males. Implants placed at 0-45^0^ had a higher failure rate (~40%) than those at 45-90° (~30%). Shorter implants (12 mm) had a failure rate of ~35%, whereas longer implants (14 mm) had a ~25% failure rate. The immediate and delayed loading protocols showed similar failure risks (~30%). Inflammation (~55% risk of failure) and mobility (~90% risk of failure) were the most significant predictors of implant failure. Poor oral hygiene was strongly associated with treatment failure (OR = 0.06). A bone density below 914 Hounsfield units (HU) was linked to higher failure rates, although ROC analysis indicated a moderate predictive ability.

Conclusion: IZC implant failure is multifactorial, with mobility, inflammation, and poor oral hygiene emerging as critical risk factors. Although bone density played a role, implant placement characteristics significantly influenced success. Clinicians should prioritize optimal insertion angles (45-90^0^), longer implants (14 mm), and strict peri-implant hygiene.

## Introduction

Orthodontic interventions frequently require supplementary anchorage control to facilitate effective and efficient tooth displacement. Traditional anchorage techniques, including extraoral headgear and intraoral appliances, are hindered by various limitations, including patient adherence and biomechanical restrictions [1]. In recent years, the advent of skeletal anchorage utilizing temporary anchorage devices (TADs) has transformed orthodontic practices by offering dependable and predictable anchorage with minimal reliance on patient compliance [2]. Among these innovations, infrazygomatic crestal orthodontic implants (IZCs) have garnered considerable acclaim due to their enhanced anchorage potential, particularly in clinical scenarios that require significant maxillary retraction, intrusion of the posterior dentition, and distalization of the maxillary arch [3,4].

IZCs are strategically placed in the dense cortical bone of the IZC, offering a high resistance to orthodontic forces. This anatomical site provides an ideal balance between accessibility and biomechanical stability, making IZCs a preferred choice for skeletal anchorage in various clinical scenarios [4]. However, despite their advantages, the failure of these implants remains a concern for orthodontists. Understanding the factors associated with treatment failure is crucial for optimizing clinical outcomes and improving patient care.

The failure rate of IZCs has been reported to be 28.1% in a study by Gill et al. [5] and 21.8% in a study by Uribe et al. [6]. Several factors contribute to the failure of infrazygomatic implants, including anatomical variations, high mandibular plane angle, surgical technique, immediate loading, insertion angle, and patient-related factors such as oral hygiene and systemic health conditions [5,7]. Although some studies have identified specific risk factors, a consensus on the primary determinants of failure remains lacking, necessitating further prospective investigations.

One of the primary factors contributing to the failure of dental implants is the insufficient primary stability at the time of implantation. Despite being composed of dense cortical bone, the infrazygomatic region may demonstrate variability in both thickness and bone density, which can influence mechanical retention of the implant [8]. Furthermore, suboptimal placement techniques, excessive mechanical loading, and inflammation of peri-implant soft tissues can undermine implant stability over time [6]. The influence of systemic conditions, including diabetes mellitus and osteoporosis, on bone remodeling and osseointegration constitutes an area of increasing scholarly interest.

Another significant factor influencing IZC success is implant design. Variations in implant diameter, length, and surface characteristics can affect biomechanical stability and long-term retention [9]. However, research suggests that the failure rate of IZCs is not associated with implant diameter or angulation [5]. The optimal implant dimensions for the infrazygomatic region have yet to be standardized. Furthermore, patient-specific factors such as age, sex, and parafunctional habits may play a role in implant longevity. Younger patients with actively remodeled bone might experience higher failure rates due to bone turnover dynamics [10]. However, Gopal et al. [11] reported no difference in the success rate of IZCs between adolescents and young adults. Patients with poor oral hygiene or habits that exert excessive force on the implant are also at an increased risk of complications [5].

Given these multifactorial influences, a comprehensive prospective study is warranted to evaluate the failure rates and factors associated with IZCs. Therefore, this study aimed to assess the failure rate of IZCs and identify key factors associated with the failure rate, providing valuable insights for clinicians to enhance treatment planning, implant selection, and surgical techniques. By understanding these variables, orthodontists can improve the predictability of IZC placement and minimize complications, ultimately leading to improved patient outcomes.

## Materials and methods

This prospective, cross-sectional, observational study was conducted in the Department of Orthodontics, Kothiwal Dental College and Research Centre, Moradabad, India, from August 2022 to December 2024 to evaluate the failure rates and associated factors influencing IZCs. Ethical clearance was obtained from the institutional review board (KDCRC/IERB/05/2022/SH21), and informed consent was obtained from all participating patients prior to enrolment in the study. This study adhered to the principles of the Declaration of Helsinki.

Patient selection and grouping

The study included systemically healthy patients with skeletal class I malocclusion, requiring IZCs for orthodontic anchorage with good oral hygiene (gingival index (GI) and plaque index (PI) < 1) [12]. Patients with systemic conditions affecting bone metabolism, such as osteoporosis or uncontrolled diabetes, those with a history of orthodontic treatment, those who were taking drugs that could affect bone metabolism, such as corticosteroids, pregnant and lactating females, those with a history of radiation therapy, any surgical procedure of the maxilla, or trauma were excluded to minimize confounding variables. The selected patients were categorized based on age and sex to assess the demographic influences on implant success rates. Age groups were stratified into adolescent (12-18 years) and adult (19-30 years) groups for comparative analysis.

Sample size estimation

Sample size estimation was performed using G*Power software version 3.6.9.1 (Heinrich-Heine-Universität Düsseldorf, Düsseldorf, Germany) to achieve a statistical power of 85% with an alpha error of 5%. The calculation was based on an effect size of 0.17, as reported in a previous study [6] that investigated the failure rate of IZCs as 21.8%. These parameters were applied in a one-sample binomial test for an expected success rate of 56%. The a priori computation yielded a sample size of 60.

Surgical procedure and implant placement

**Figure 1:**
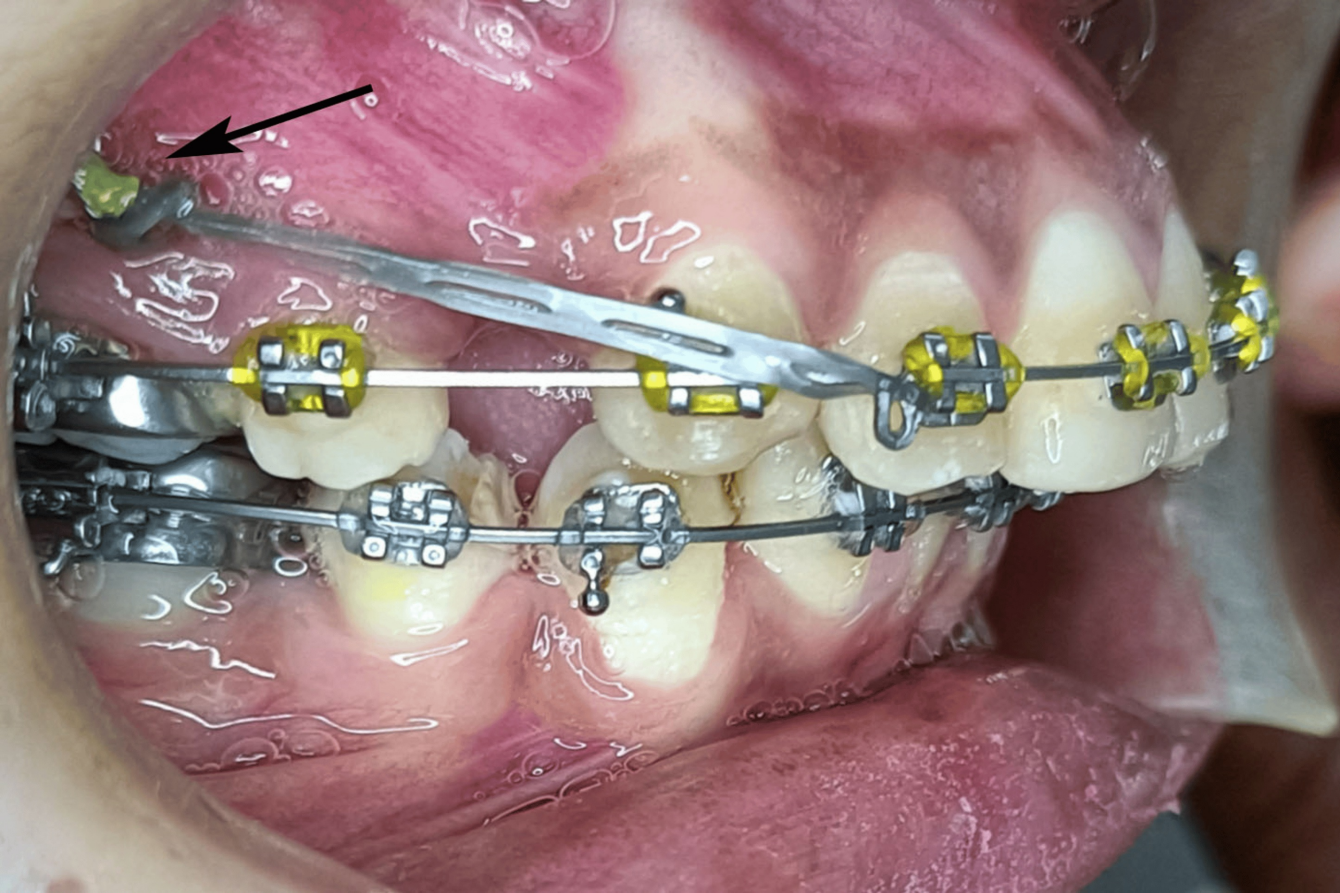
Infrazygomatic implant placed in the patient. This figure is of a patient from the study and used with patient's permission.

All IZCs (Dentos Absoanchor, Dentos India Pvt. Ltd., Mumbai, India) of length 12/14 mm, and a diameter of 2 mm were placed at a height of 14-16 mm from the maxillary occlusal plane at an initial insertion angle of 90^0^, gradually changing to 55-70^0^ by a single experienced orthodontist (SG) using a standardized surgical protocol to reduce operator-related variability (Figure 1).

The implants were inserted into the IZC following the administration of local anesthesia. The angle of insertion was carefully measured and recorded using cone-beam computed tomography (CBCT), considering its impact on primary stability and implant longevity.

Assessment of bone density

The bone density at the IZC was evaluated using CBCT before implant placement by two calibrated examiners (PP, KR) independently. CBCT scans provided a quantitative assessment of bone quality. The influence of bone density on implant stability and failure rates was analyzed postoperatively.

Loading protocols and stability analysis

This study compared immediate and delayed loading protocols to determine their effects on implant survival. In the immediate loading group, orthodontic forces of 200-400 grams measured by Dontrix gauge (GDC Fine Crafted Dental Pvt. Ltd., India) depending on the treatment mechanics (enmass retraction of anterior teeth in cases of dental Class II Division I malocclusion or Class I bimaxillary protrusion treated with extraction approach, intrusion of posterior teeth in case of skeletal open bite, distalization of maxillary arch in cases of dental Class II Division I malocclusion treated with non-extraction approach), were applied within one week of placement, whereas in the delayed loading group, force application was deferred for at least four weeks to allow for implant stabilization. Implant stability was assessed at regular intervals using resonance frequency analysis and clinical examination.

Peri-implantitis and oral hygiene evaluation

The presence of peri-implantitis was monitored using clinical signs of inflammation. The patients were instructed on oral hygiene maintenance, and their compliance was evaluated periodically. Deterioration of oral hygiene during the treatment was noted as a potential risk factor for implant failure, as assessed using PI and GI.

Other factors

In all cases, bilateral IZCs were placed, and the side of failure was noted to determine if the side of placement influenced the outcomes. Factors such as dominant chewing side and asymmetrical occlusal forces were considered in the analysis.

Follow-up and reliability testing

The principal outcome of the study was failure of the IZC. The term failure was operationally defined as the removal or loss of the mini screw occurring within a period of less than eight months post-implantation (early and late failures). The patients were followed up for a minimum of 12 months post-implantation. All assessments were performed by two calibrated examiners, and measurements were repeated after two weeks to assess intra- and inter-examiner reliability using the intraclass correlation coefficient (ICC).

Statistical analysis

Statistical analyses were conducted using SPSS software version 23 (IBM Corp., Armonk, NY, USA) by a statistician (VK) who was provided with coded data. The normality of the data was assessed using the Kolmogorov-Smirnov test, which revealed that age and bone density exhibited a non-normal distribution. Data are reported as absolute frequencies and percentages. Continuous variables, including age, bone density, and follow-up period, are expressed as mean and standard deviation. Univariate regression analysis was used to determine the odds ratio (OR) and risk ratio for each predictor. The Youden Index was calculated to evaluate the predictive ability of bone density for success, accompanied by a receiver operating characteristic (ROC) curve to establish a cut-off value. A significance level of p < 0.05 was adopted as the threshold.

## Results

The ICC values of 0.82 for inter-examiner, and 0.86 for intra-examiner reliability showed good reliability and reproducibility. Of these 60 implants, 18 (30%) failed. The failure rates of the IZCs varied across the different parameters. Among the age groups, individuals older than 18 years had a higher failure rate than those aged < 18. Females experienced a higher failure rate than males. Implants placed at-0-45^0^ angle showed a higher failure rate than those placed at 45-90^0^. Immediate loading resulted in a failure rate, whereas delayed loading resulted in a slightly lower failure rate. The presence of inflammation significantly increased the failure rate compared to that in cases without inflammation. Similarly, mobility was a strong predictor of failure, with mobile implants showing a high failure rate, whereas stable implants had a much lower failure rate. Implants of 12 mm length had a higher failure rate than 14 mm implants. The left side had a higher failure rate than the right side. Poor oral hygiene was strongly associated with implant failure, whereas good oral hygiene was significantly associated with a lower failure rate. These findings suggest that factors, such as inflammation, mobility, poor oral hygiene, and shorter implant length, contribute to higher failure rates (Table 1).

**Table 1:** Absolute frequency and failure rate of infrazygomatic crest (IZC) implants. Data are presented in the form of frequency and percentage.

Parameters	Category	Total/failed	Failure rate
Age group in years	<18	28/6	21.43%
	>18	32/12	37.50%
Sex	Male	32/9	28.13%
	Female	18/9	50.00%
Implant angle in degrees	0-45	10/4	40.00%
	45-90	50/14	28.00%
Loading	Immediate	46/14	30.43%
	Delayed	14/4	28.57%
Inflammation	Yes	31/17	54.84%
	No	29/1	3.45%
Mobility	Yes	11/10	90.91%
	No	49/8	16.33%
Implant length in mm	12	29/11	37.93%
	14	31/7	22.58%
Side	Right	34/6	17.65%
	Left	26/12	46.15%
Oral hygiene	Poor	19/13	68.42%
	Good	41/5	12.20%

The mean age of the surviving group was 18.9 years, whereas the failed group had a slightly higher mean age of 19.89 years (p = 0.372), indicating no statistically significant difference. The bone density was lower in the failed group (994.89 HU) than in the surviving group (1,080.98 HU), suggesting a trend toward significance. These results imply that while age did not significantly affect IZC survival, lower bone density may be associated with a higher risk of failure (Table 2).

**Table 2:** Mann-Whitney U test to compare continuous parameters in groups of infrazygomatic crest (IZC) implants. Data are presented in the form of mean and standard deviation (SD). HU: Hounsfield units, p-value < 0.05: significant.

Parameters	Unit	Survived		Failed		Z-value	P-value
		Mean	SD	Mean	SD		
Age	years	18.9	3.47	19.89	3.5	-0.89	0.372
Bone density	HU	1080.98	154.31	994.89	129.25	-1.88	0.06*

The OR analysis revealed the key factors influencing IZC failure. Individuals under 18 years of age had higher odds of survival (OR = 2.2), although the confidence interval suggests variability. Males had higher odds of survival than females (OR = 2.56). The implant angle (0-45^0^) showed a lower OR (0.58), indicating a potential association with failure. Immediate loading had an OR close to 1 (OR = 0.91), suggesting no significant impact. Inflammation dramatically increased the odds of failure (OR = 0.82), whereas mobility was strongly associated with failure (OR = 0.11), indicating a high likelihood of survival in non-mobile implants. A 12-mm implant length had lower odds of survival (OR = 0.47) compared to 14 mm. Right-sided placement showed higher odds of survival (OR = 2.33). Notably, poor oral hygiene was strongly linked to failure (OR = 0.06), emphasizing its critical role in IZC success. These findings highlight mobility and oral hygiene as the most influential factors for IZC survival (Table 3).

**Table 3:** Independent association between predictor variables and failed implants. Data are presented in the form of n (%).

Parameters	Category	Survived		Failed		Odds ratio	Confidence interval 95%
		n	%	n	%		
Age group in years	<18	22	52.38	6	33.33	2.2	0.7 - 6.96
	>18	20	47.62	12	66.67		
Sex	Male	23	54.76	9	50.00	2.56	0.4 - 3.66
	Female	9	21.43	9	50.00		
Implant angle in degrees	0-45	6	14.29	4	22.22	0.58	0.14 - 2.38
	45-90	36	85.71	14	77.78		
Loading	Immediate	32	76.19	14	77.78	0.91	0.24 - 3.42
	Delayed	10	23.81	4	22.22		
Inflammation	Yes	14	33.33	17	94.44	0.82	0.41 - 1.67
	No	28	66.67	1	5.56		
Mobility	Yes	1	2.38	10	55.56	0.11	0.07 - 0.37
	No	41	97.62	8	44.44		
Implant length in mm	12	18	42.86	11	61.11	0.47	0.14 - 1.34
	14	24	57.14	7	38.89		
Side	Right	28	66.67	6	33.33	2.33	0.18 - 1.29
	Left	14	33.33	12	66.67		
Oral hygiene	Poor	6	14.29	13	72.22	0.06	0.03 - 0.21
	Good	36	85.71	5	27.78		

The risk of implant failure varies depending on several factors. Patients under 18 years of age had a lower failure risk (~25%) by six months compared to those above 18 (~35%). Implants placed at 0-45^0^ show a higher risk of failure (~40%) than those at 45-90^0^ (~30%). Similarly, shorter implants (12 mm) had a greater failure risk (~35%) than longer implants (14 mm, ~25%). Delayed and immediate loading both resulted in a failure risk of approximately 30%. The presence of inflammation (~55% risk) and implant mobility (~90% risk) drastically increased the failure rates, making them the most critical risk factors. In contrast, stable implants and the absence of inflammation significantly enhanced implant survival (Figure 1).

**Figure 2:**
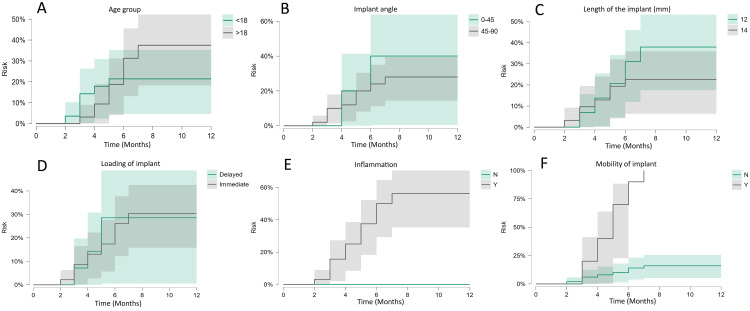
Risk analysis of predictor variables with respect to time in months. (A) Age group, (B) implant insertion angle (0-90 degrees), (C) length of implant (mm), (D) loading of implant, (E) inflammation, and (F) mobility of implant. This figure is based on the data obtained from this study.

**Figure 3:**
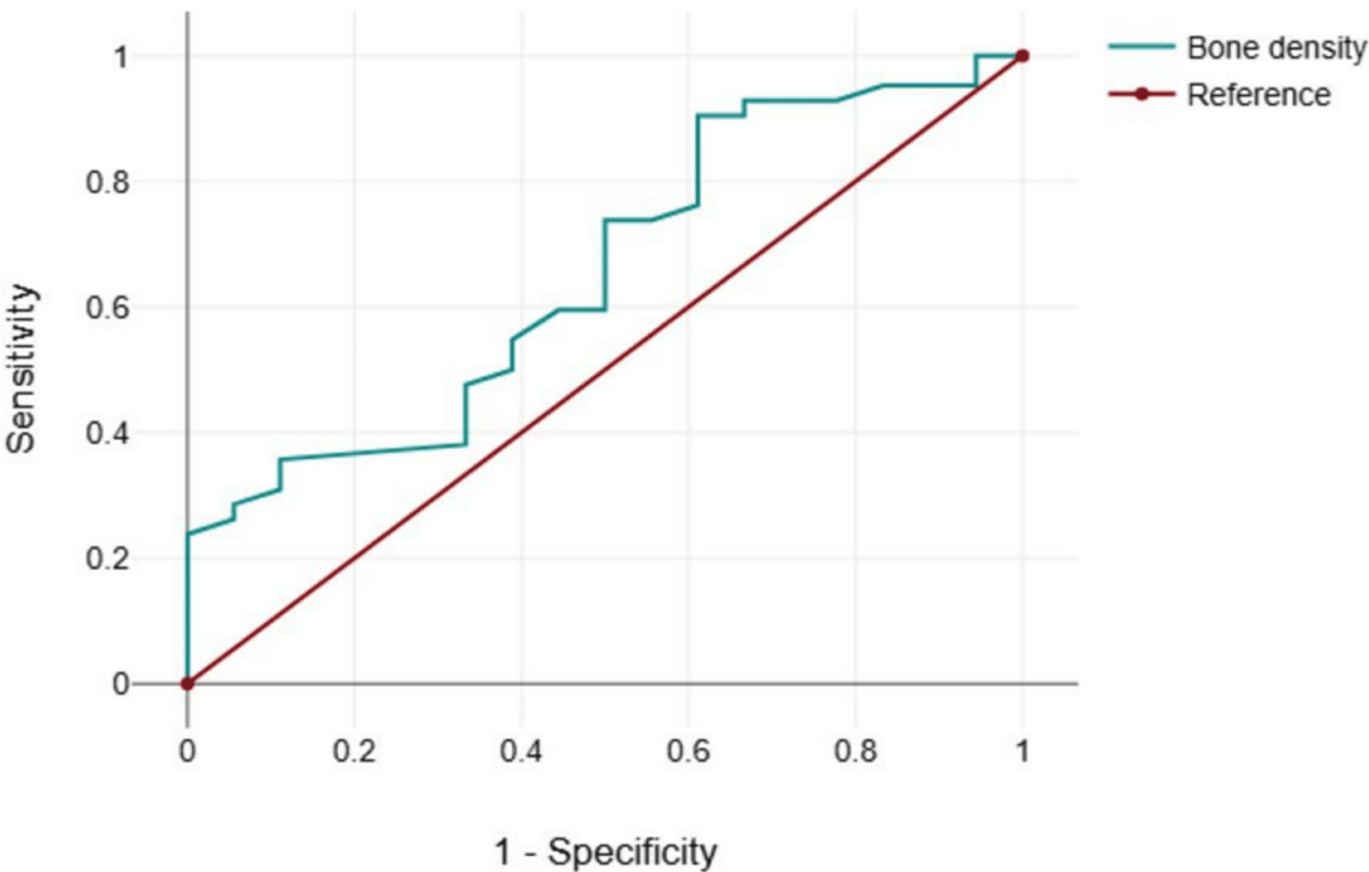
Receiver operating characteristic (ROC) analysis for bone density. This figure is based on the data obtained from this study. AUC: 0.654

ROC analysis for bone density as a predictor of IZC success showed an area under the curve (AUC) of 0.654, indicating moderate predictive ability. A cut off value of 914 HU yielded a sensitivity of 90.5%, indicating that it correctly identified the most successful cases, while the false-positive rate (1 - specificity) was 61.1%. The Youden index was 0.294, suggesting limited but notable discriminatory power. These results imply that bone density plays a role in predicting IZC success, with a threshold of 914 HU optimizing sensitivity at the cost of false positives (Table 4, Figures 2A-2F, 3).

**Table 4:** Receiver operating characteristic (ROC) analysis and cut-off value of bone density in Hounsfield units (HU) for success prediction. AUC: area under the curve

Bone density	AUC	Sensitivity	1 - Specificity	Youden index
914	0.654	0.905	0.611	0.294

## Discussion

This study aimed to evaluate the failure rates and associated factors influencing IZC implants in orthodontic anchorage. These findings suggest that multiple factors, including patient demographics, implant placement characteristics, loading protocols, bone density, and oral hygiene, contribute significantly to the success or failure of IZCs. This study provides valuable insights into the clinical considerations for optimizing IZC survival and reducing failure risks. The failure rate of IZC was 30% in our study, which was in accordance with a study by Gill et al. [5], who reported a failure rate of 28.1%.

Age and sex influence on IZC failure

The results indicated that patients aged > 18 exhibited a higher failure rate than those aged < 18. This suggests that, although older age may present an increased risk of implant failure, it is not a definitive predictor. However, age was significantly associated with IZC failure. Similar results have been reported by Gill et al. [5]. This finding indicates that the bone remodeling capacity declines with age, potentially impacting implant stability [12].

Sex differences were also noted, with females exhibiting a higher failure rate than males. The OR for male survival was 2.56, suggesting that males had a higher probability of IZC success. Hormonal variations, bone density differences, and occlusal force disparities between males and females may have contributed to this trend [13]. However, further research is required to explore these associations in greater depth.

Implant placement angle and length

The study found that implants placed at-0-45^0^ angle had a higher failure rate than those placed at 45-90^0^. The OR for survival at lower angles was 0.58, indicating that steeper angles may improve implant stability. This could be attributed to increased cortical bone engagement at higher angles, which enhances primary stability. Previous studies have corroborated this finding, suggesting that steeper insertion angles reduce micromotion and enhance osseointegration [10]. Pan et al. [14] reported the highest bone density at insertion angles of 70^0^ and 17 mm above the maxillary occlusal plane.

Similarly, implant length plays a significant role in failure rates. Shorter implants (12 mm) had a higher failure rate than did longer implants (14 mm). The OR for 12 mm implants was 0.47, indicating reduced survival. Longer implants result in a greater surface area of the bone, enhancing mechanical stability and reducing the likelihood of early failure [15]. These findings underscore the importance of selecting appropriate implant dimensions to optimize IZC longevity. Tseng et al. [16] identified a comprehensive success rate of 91.1% associated with mini-screws, while achieving a success rate of 100% for mini-screws ≥ 12 mm. Their findings indicated that the success rate increased with an increase in the mini-screw length.

Loading protocols: immediate vs. delayed

The study compared immediate and delayed loading protocols, revealing a failure risk of approximately 30% in both groups. The OR for immediate loading was 0.91, suggesting no significant impact on failure rates. This was in accordance with the study by Costa et al. [17]. In contrast, some studies have suggested that immediate loading may compromise stability owing to insufficient osseointegration [13,18]. However, controlled force application in immediate loading cases may mitigate these risks. The findings indicate that both protocols are viable, provided adequate primary stability is achieved during placement.

Impact of inflammation and mobility

Inflammation has emerged as a critical factor, with affected patients exhibiting a significantly higher risk of failure than those without inflammation. The OR for inflammation-related failure was 0.82, reinforcing its strong association with implant loss. Peri-implantitis, characterized by progressive bone loss and soft tissue inflammation, is a well-documented cause of implant failure [19]. Effective peri-implant care and strict oral hygiene protocols are essential to minimize inflammation-related failures. As stated by Chang et al. [20], it is imperative to ensure a minimum clearance of 5 mm between the head of the bone screw and the surrounding soft tissue to promote optimal oral hygiene practices and aid in the management of peri-screw inflammation. Inflammatory processes adversely affect the bone surrounding the neck of the bone screws, and ongoing deterioration of the cortical bone leads to instability and potential loss of the implant.

Mobility is another key predictor of failure, with mobile implants exhibiting an exceptionally high failure rate. The OR for stable implants was 0.11, indicating a strong likelihood of survival in the absence of mobility. Primary stability at the time of placement is crucial in determining long-term success because excessive micromotion can disrupt osseointegration. These findings highlight the need for careful selection of surgical techniques and patients to ensure implant stability. Liou et al. [21] conducted a focused examination of the IZC, revealing that this category of screws exhibits a certain level of mobility without leading to failure. Nonetheless, our findings indicate that such mobility is significantly correlated with the instances of failure.

Oral hygiene and bone density

Poor oral hygiene was strongly associated with implant failure with an OR of 0.06, emphasizing its critical role in IZC survival. Plaque accumulation and inadequate hygiene can predispose patients to peri-implantitis, thereby increasing the likelihood of failure. Patients with good oral hygiene exhibited significantly lower failure rates, reinforcing the importance of patient education and compliance for maintaining implant health. This finding is supported by previous studies by Gill et al. [5], Uribe et al. [6], and Sharma et al. [22].

Bone density was another influential factor, with failed implants showing lower density values (994.89 HU) than surviving implants (1,080.98 HU). ROC analysis indicated moderate predictive ability. These findings suggest that while bone density assessment can guide implant planning, it should be considered along with other clinical parameters [8]. Findings derived from research involving animal models suggest that the mobility of mini implants exhibits a negative correlation with the torque applied during insertion, while demonstrating a positive correlation with both the density of bone mineral content and thickness of the cortical bone [23].

Side of placement and occlusal forces

A notable observation was the higher failure rate on the left side than on the right. The OR for right-sided survival was 2.33, suggesting a significant difference. This could be attributed to variations in occlusal forces, dominant chewing side, or anatomical differences in the bone structure [24]. Although further research is needed to confirm these associations, clinicians should consider such asymmetries during treatment planning.

Clinical implications of the study

The findings of this study have significant clinical implications for orthodontists who utilize IZCs. Optimizing implant placement by preferring a steeper insertion angle (45-90^0^) and longer implant length (14 mm) can enhance stability. Managing inflammation and minimizing mobility during placement through strict peri-implant hygiene are critical factors for success. Although bone density influences outcomes, it should be used in conjunction with other clinical factors to guide treatment decisions. Additionally, both immediate and delayed loading protocols are viable options if adequate stability is ensured.

Limitations and future recommendations

Although this study provides valuable insights into the factors influencing IZC failure, certain limitations should be acknowledged. A sample size of 60, although statistically justified, limits the generalizability of the findings. Additionally, a follow-up period of 12 months, which is sufficient for assessing early and late failures, may not capture the long-term trends in IZC survival. Future research should explore larger multicenter studies with extended follow-up durations to validate these findings. Another limitation is the potential influence of unmeasured variables such as patient compliance with oral hygiene recommendations and individual variations in masticatory forces, growth patterns, and skeletal patterns. Further studies incorporating multivariate statistical modelling and artificial intelligence-based predictive algorithms may enhance our understanding of the success and failure mechanisms of IZC.

## Conclusions

This study highlights the multifactorial nature of IZC failure, emphasizing the role of age, implant placement characteristics, inflammation, mobility, oral hygiene, and bone density. Among these, mobility and inflammation are the most significant predictors of failure. Older age, implants placed at 0-45^0^, and shorter implants (12 mm) were other risk factors. Both delayed and immediate loading result in an equal failure risk. The failure rate of the IZC was found to be 30%. Future studies with larger sample sizes and longer follow-up periods are warranted to refine these findings and improve the clinical outcomes of orthodontic anchorage procedures.
